# Optimization of gearbox housing shape for agricultural UTV using structural–acoustic coupled analysis

**DOI:** 10.1038/s41598-024-54606-8

**Published:** 2024-02-20

**Authors:** Beom-Soo Kim, Hyun-Woo Han, Woo-Jin Chung, Young-Jun Park

**Affiliations:** 1 Department of Biosystems Engineering, College of Agriculture and Life Science, Seoul National University, Seoul, South Korea; 2 Convergence Major in Global Smart Farm, College of Agriculture and Life Sciences, Seoul National University, Seoul, South Korea; 3 Research Institute of Agriculture and Life Sciences, Seoul National University, Seoul, South Korea

**Keywords:** Mechanical engineering, Engineering, Software

## Abstract

In this study, gearbox radiated noise was successfully reduced through housing shape optimization. First, dynamic and structural–acoustic coupled analysis models of an agricultural UTV gearbox were developed. Then, the test equipment was configured to match that of the simulation model, and a test was conducted. The analysis and test results showed errors within 0.1 dB for vibration and 0.2 dB(A) for noise, indicating that the models were reliable. The housing design was then optimized using topology optimization based on the developed structural–acoustic coupling analysis model. The sound pressure level around the housing was used as an objective function for topology optimization. The optimal distribution of materials for the housing was also derived to reduce the radiated noise. Lastly, the housing rib was designed based on the optimization result, and an improvement in the radiated noise by approximately 2.43 dB(A) was confirmed in the operation area.

## Introduction

Recently, in addition to large agricultural machines such as tractors and combines, research on multi-purpose agricultural vehicles, such as utility terrain vehicles (UTVs), has been actively conducted. Many agricultural UTVs are equipped with a rollover protection structure (ROPS) rather than a cabin. Their simple structure lacks sound absorbing and insulation elements; therefore, UTV drivers are directly exposed to the noise generated by the powertrain.

The electrification of agricultural UTVs is progressing faster than that of other large agricultural machines since they require a relatively small amount of power for agricultural work and have a simple powertrain^1^. The noise of the motor and gearbox is the primary source of noise in an electrified powertrain^2^. In particular, the most prominent noise generated from a gearbox is gear noise, which can be detected by human ears even when the pure noise of the gear is approximately 10 dB(A) lower than the overall noise level^3^.

Elastic deformation of gear teeth and factors such as manufacturing and assembly errors in gears lead to variations in mesh stiffness during the rotation of gears. Mesh stiffness variations and misalignment of gears during power transmission of a gearbox causes a gear transmission error, which acts as an excitation source for the gearbox^4,5^. Vibration due to gear excitation is transmitted to the gearbox housing through the gear blank, shaft, and bearing and is radiated from the housing surface in the form of noise. The noise from the housing is reduced when the vibration of the gearbox housing is reduced. However, depending on the shape and radiation efficiency of the housing, in some cases a decrease in the vibration in a specific frequency range can result in an increase in noise^6,7^. Therefore, the vibration and vibro-acoustic behaviour of the gearbox and the noise propagation medium must be studied to accurately predict the radiated noise of the gearbox housing^8,9^.

Tanaka et al. calculated the housing vibration by inputting the gear and bearing vibration results derived using an in-house code into a housing finite element model^10^. They presented a method for predicting the radiated noise of a two-stage helical gearbox by inputting the calculated housing vibration into the BEM analysis model. Guo et al. used a lumped-parameter model that included gears, bearings, and shafts to obtain housing vibration levels in a simpler manner^11^. Subsequently, they presented a method for predicting radiated noise by inputting the derived housing vibration into the BEM model. Mordillat et al. presented a method for predicting the radiated noise of a powertrain through finite element analysis^12^. They presented a method to obtain radiated noise with a small amount of computation using a reduction method on their finite element analysis model.

These studies all developed analysis models to predict the vibration and noise of housing using various methods. Noise and vibration tests were then performed to verify the reliability of the developed model. However, the gearbox and various elements such as the testbed, jig, motor, coupling, and torquemeter are included in the test to verify this analysis model. Since these factors affect the boundary condition of the gearbox and change the dynamic excitation force of the gear, the dynamic characteristics of all the elements used in the test must be considered in the analysis model to develop an accurate model. As most previous studies have only considered the gearbox unit in their analysis models, without considering the mass moment of inertia, stiffness of the test device, or systems other than the gearbox, their results may differ from the dynamic characteristics recorded in an actual test. Therefore, in this study, we developed a more reliable model by including all factors used in the model validation test in the boundary conditions of the analysis model.

Ogata et al. used topology and shape optimization techniques to design transmission gearboxes with high stiffness^13^. After selecting the positions of the ribs and reinforcing materials using the topology optimization results, the ribs were designed. Then, shape optimization was used to determine the optimal thickness of the ribs, from which the shape of the housing with reduced weight was derived. This also satisfied the stiffness criterion. Ide et al. optimized the housing shape using the radiation noise prediction method based on finite element and boundary element analyses and the topology optimization method^14^. After analyzing the housing vibration using finite element analysis, they entered the vibration results into the boundary element to excite the acoustic space. Phase optimization conditions that can minimize both the housing weight and sound pressure at a single point were presented. Thus, they derived a housing shape that can reduce both the weight and radiated noise. Son et al. used a topology optimization method to reduce the radiated noise of a housing^15^. The equivalent radiated power (ERP) was used as an objective function for the optimization, and the shape of the housing with reduced ERP was derived.

Many previous studies related to housing optimization have not validated the model used for optimization. In addition, since variables such as ERP do not consider radiation efficiency, they tend to overestimate the noise level at low frequencies. Therefore, such variables are not appropriate as variables for optimizing the radiation noise of the actual housing. In this study, accurate noise prediction was performed using the structural–acoustic coupled analysis for the experimentally verified model. We aim to find an optimized shape for a more reliable housing using the sound pressure level (SPL) as an objective function.

**Figure 1:**
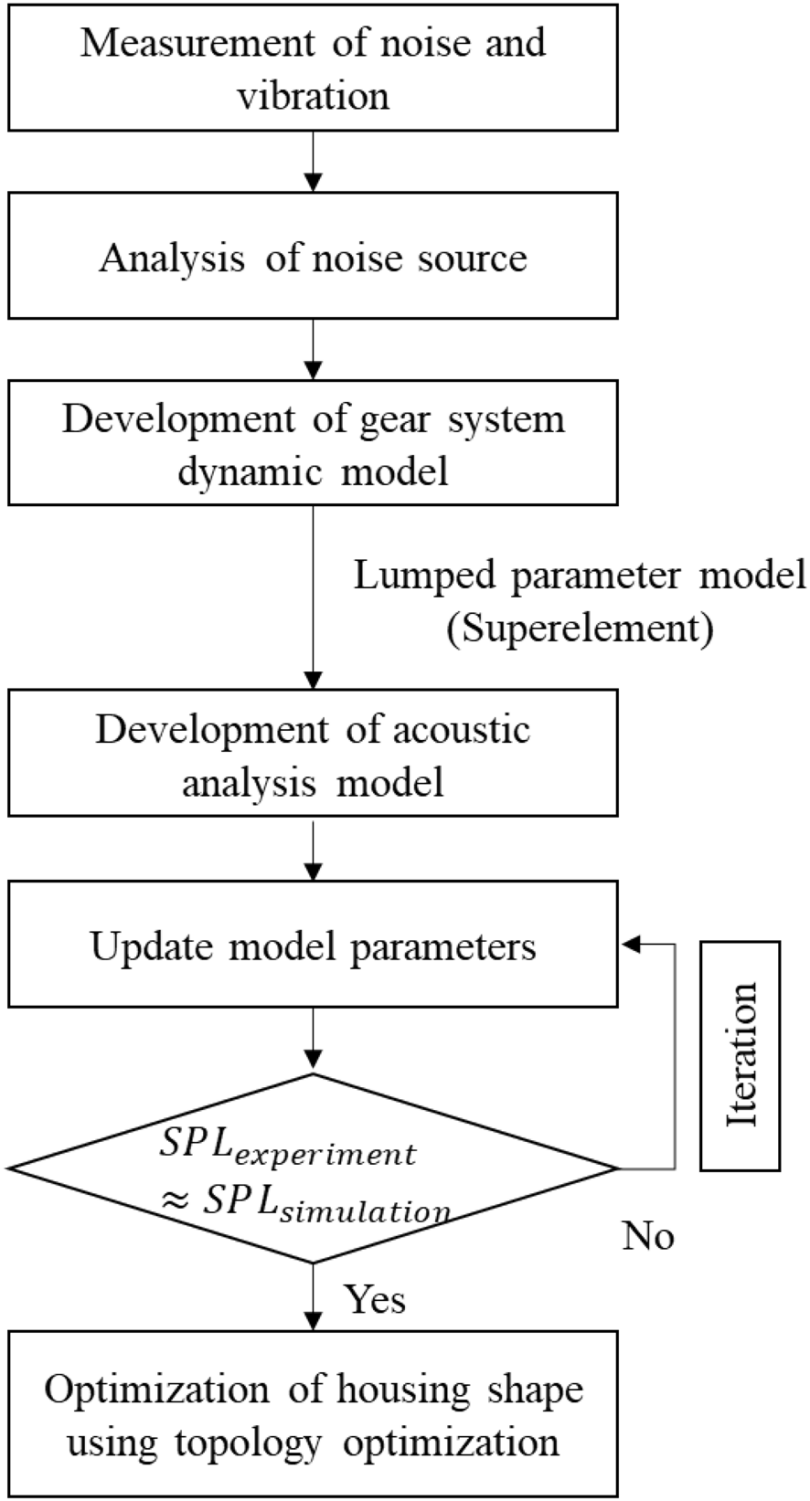
Flowchart for the proposed optimization of the shape of the gearbox housing.

In summary, we developed a simulation model by considering the majority of factors used in the verification test to supplement the limitations of the existing gearbox radiation noise prediction research. We developed a simulation model that included the gearbox and all the elements that are necessary for driving the gearbox. Certainly, the simulation model did not achieve an impeccable implementation of all the elements of the gearbox used in the verification experiment. However, since the aim of this study was to examine the feasibility of an analytical design approach through new design criteria, the focus was not on creating a perfect model, but rather on constructing a model at a level suitable for the experimental environment. Assumptions were made where necessary, aiming to create a model that exhibited behaviour as close as possible to the test results at a feasible level. A more accurate excitation force due to gear meshing was implemented in the model. In addition, a test verification of the simulation model was also performed; an accurate gear excitation force was realized in this study. This posed a limitation in many previous studies related to the optimization of the shape of the existing gearbox housing. Finally, when the shape of the housing was optimized using the verified model, more reliable optimization results were obtained using sound pressure considering radiation efficiency as an objective function. Thus, our study overcomes some of the limitations of previously published results. Figure 1 schematically illustrates the process of our approach.

## Gearbox test design

**Figure 2:**
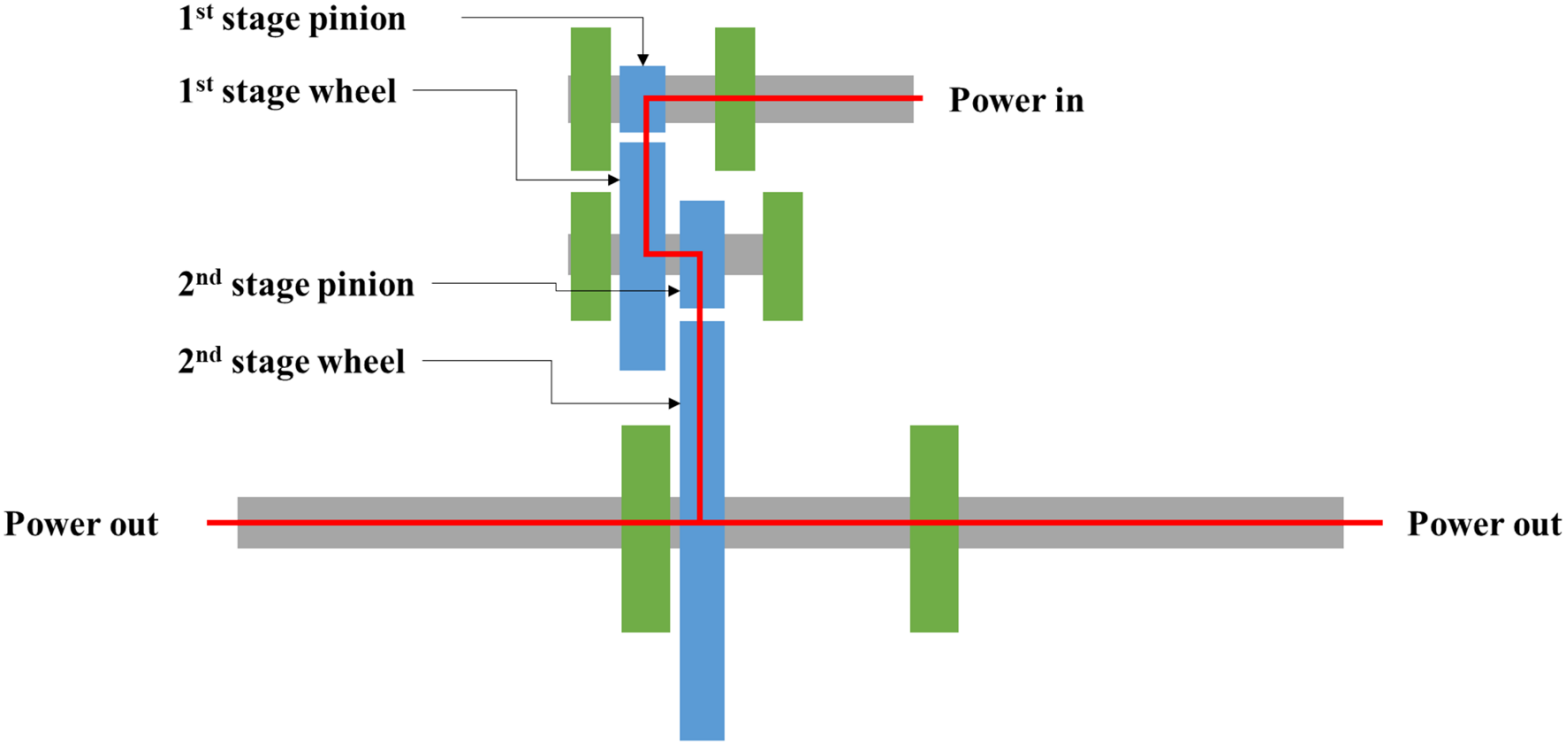
Schematic diagram of target UTV gearbox.

The target gearbox used in this study was a reduction gearbox mounted on the drive motor of an agricultural UTV. This gearbox consists of a two-stage helical gear and differential gear, receives power from a 7 kW drive motor, and transmits power to the wheels of the vehicle attached to both ends of the output shaft. The gearbox housing was made of aluminium and fixed to the vehicle using bolts. The gear specifications used in the gearbox are listed in Table 1, and the gearbox structure is shown schematically in Fig. 2. Originally, the gearbox received power at a rated input speed of 2650 rpm and transmitted power to the wheels through the differential gear set. However, in this study, the differential gear set was removed for the convenience of analysis and testing.

**Table 1:** Gear specification of target UTV gearbox.

Parameter	1st stage		2nd stage	
	Pinion	Wheel	Pinion	Wheel
Number of teeth	14	46	21	79
Face width, mm	16	16	18	16
Normal module, mm	1.75		1.75	
Pressure angle, deg	20		20	
Helix angle, deg	25		25	
Centre distance, mm	57		97	
Profile shift coefficient	0.2	− 0.7299	0.2	0.0597

**Figure 3:**
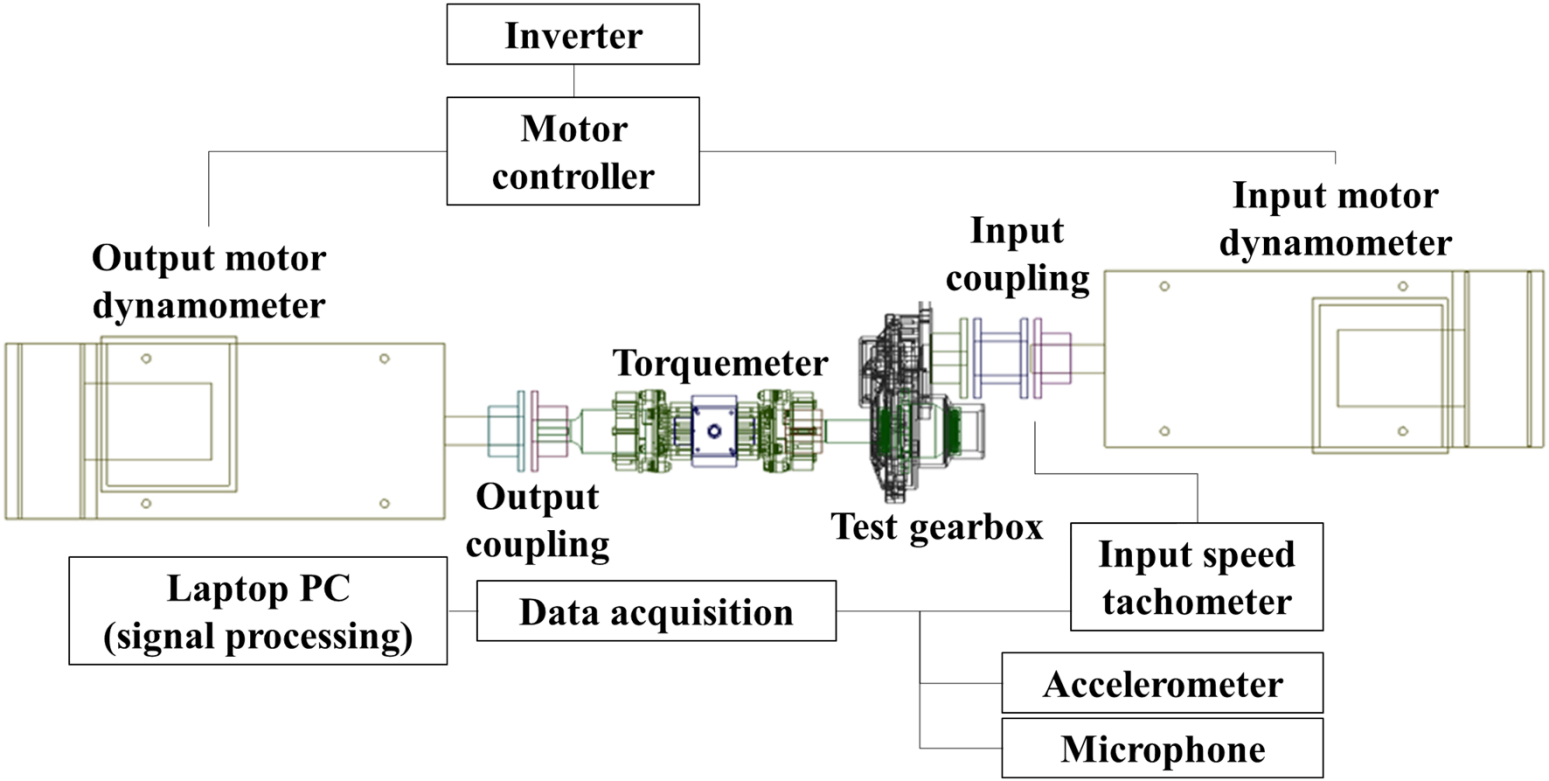
2D diagram of gearbox test.

A back-to-back motor dynamometer was used to measure the vibration and noise generated in the gearbox. An inverter and motor dynamometer were used to control the rotation speed of the input shaft and the load torque of the output shaft. The power of the input dynamometer was transmitted to the gearbox and the output dynamometer through flexible coupling. A torquemeter was connected to measure the load on the output shaft. The test equipment is illustrated in Fig. 3. Table 2 lists the elements that were used in the test that can affect the dynamic behaviour of the gearbox.

**Table 2:** Parameters derived from component catalogues and shapes.

Component	Parameter	Value
Input/output motor dynamometer	Mass moment of inertia	0.123 kg m^2^
	Mass	140 kg
Input coupling	Mass	6 kg
	Torsional stiffness	45,000 kgf m/rad
Torquemeter	Mass moment of inertia	0.0072 kg m^2^
	Mass	3.5 kg
	Axial stiffness	148 N/mm
	Radial stiffness	9010 N/mm
Output coupling	Mass	10 kg
	Torsional stiffness	13,750 Nm/rad

## Development of simulation model

### Governing equation

Systems such as agricultural UTV gearboxes have complex shapes and boundary conditions. Thus, predicting the noise and vibration of the systems through theoretical solutions is very difficult. Many studies use numerical solutions involving finite elements to obtain approximate solutions for noise and vibration. In this case, the approximate solution can be obtained by solving the governing equations of the system, expressed by Eqs. (1) and (2), in the finite element model of the gearbox structure and acoustic system using numerical analysis.

1 $$\left[{{\varvec{M}}}_{S}\right]\left\{\ddot{{\varvec{w}}}\left(t\right)\right\}+\left[{{\varvec{C}}}_{S}\right]\left\{\dot{{\varvec{w}}}\left(t\right)\right\}+\left[{{\varvec{K}}}_{S}\right]\left\{{\varvec{w}}\left(t\right)\right\}=\left\{{\varvec{f}}\left(t\right)\right\}$$

2 $$\left[{{\varvec{M}}}_{F}\right]\left\{\ddot{{\varvec{p}}}\left(t\right)\right\}+\left[{{\varvec{C}}}_{F}\right]\left\{\dot{{\varvec{p}}}\left(t\right)\right\}+\left[{{\varvec{K}}}_{F}\right]\left\{{\varvec{p}}\left(t\right)\right\}=\left\{{\varvec{q}}\left(t\right)\right\}$$

Here, ${{\varvec{M}}}_{S}$, ${{\varvec{K}}}_{S}$, ${{\varvec{C}}}_{S}$ are the mass, stiffness, and damping matrices of the structural system, respectively; ${{\varvec{M}}}_{F}$, ${{\varvec{K}}}_{F}$, ${{\varvec{C}}}_{F}$ are the mass, stiffness, and damping matrices of the acoustic cavity, respectively; ${\varvec{w}}$ is the displacement vector of the system; ${\varvec{f}}$ denotes the external force vector acting on the system; ${\varvec{p}}$ denotes the pressure in the system; and ${\varvec{q}}$ denotes the acoustic excitation vector. When the acoustic and elastic structures are in contact, the pressure acting on the fluid lattice at the fluid–structure interface excites the structural region. Conversely, the pressure acting on the structural lattice excites the fluid region. Therefore, the governing equations of the structural and acoustic systems are connected and expressed as a single structural–acoustic coupled equation. This equation can be obtained by adding the relational expression at the fluid–structure interface to the equations of motion of the structural and acoustic systems derived earlier.

The equations of motion of the structural and acoustic systems, considering the effect of the fluid–structure interface, are expressed by Eqs. (3) and (4):

3 $$\left[{{\varvec{M}}}_{S}\right]\left\{\ddot{{\varvec{w}}}\left(t\right)\right\}+\left[{{\varvec{C}}}_{S}\right]\left\{\dot{{\varvec{w}}}\left(t\right)\right\}+\left[{{\varvec{K}}}_{S}\right]\left\{{\varvec{w}}\left(t\right)\right\}-\left[{\varvec{S}}\right]\left\{{\varvec{p}}\left(t\right)\right\}=\left\{{\varvec{f}}\left(t\right)\right\},$$

4 $$\left[{{\varvec{M}}}_{F}\right]\left\{\ddot{{\varvec{p}}}\left(t\right)\right\}+\left[{{\varvec{C}}}_{F}\right]\left\{\dot{{\varvec{p}}}\left(t\right)\right\}+\left[{{\varvec{K}}}_{F}\right]\left\{{\varvec{p}}\left(t\right)\right\}-\left[{\varvec{R}}\right]\left\{\ddot{{\varvec{w}}}\left(t\right)\right\}=\left\{{\varvec{q}}\left(t\right)\right\}.$$

Here, ${\varvec{R}}$ represents the acoustic-structural coupling matrix and ${\varvec{S}}$ represents the structural–acoustic coupling matrix. Each coupling matrix has a reciprocity of $\left[{\varvec{S}}\right]={\left[{\varvec{R}}\right]}^{T}$, and, by combining Eqs. (3) and (4), a structural–acoustic coupled equation can be obtained:

5 $$\left[\begin{array}{cc}{{\varvec{M}}}_{{\varvec{S}}}& 0\\ -{{\varvec{S}}}^{{\varvec{T}}}& {{\varvec{M}}}_{{\varvec{F}}}\end{array}\right]\left\{\begin{array}{c}\ddot{{\varvec{w}}}\left(t\right)\\ \ddot{{\varvec{p}}}\left(t\right)\end{array}\right\}+\left[\begin{array}{cc}{{\varvec{C}}}_{{\varvec{S}}}& 0\\ 0& {{\varvec{C}}}_{{\varvec{F}}}\end{array}\right]\left\{\begin{array}{c}\dot{{\varvec{w}}}\left(t\right)\\ \dot{{\varvec{p}}}\left(t\right)\end{array}\right\}+\left[\begin{array}{cc}{{\varvec{K}}}_{{\varvec{S}}}& -{\varvec{S}}\\ 0& {{\varvec{K}}}_{{\varvec{F}}}\end{array}\right]\left\{\begin{array}{c}{\varvec{w}}\left(t\right)\\ {\varvec{p}}\left(t\right)\end{array}\right\}=\left\{\begin{array}{c}{\varvec{f}}\left(t\right)\\ {\varvec{q}}\left(t\right)\end{array}\right\}.$$

### Vibration model

**Figure 4:**
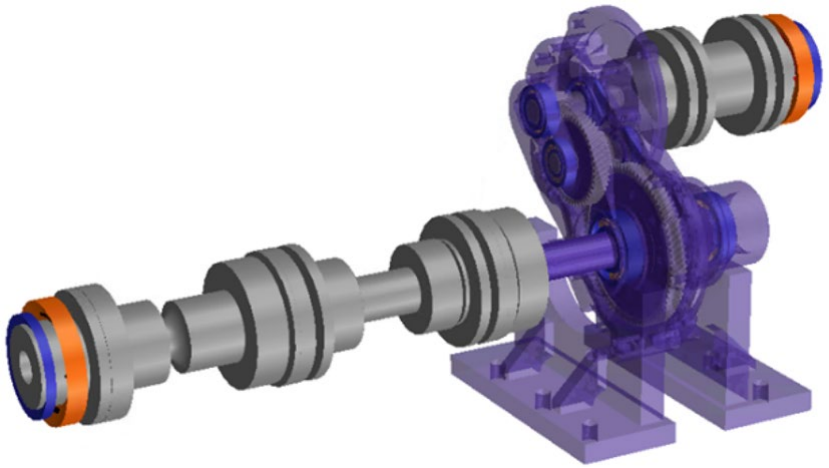
Dynamic model of the gear system.

As shown in Fig. 2, the system used in this study consists of a gearbox composed of gears, shafts, bearings, and housing, and a shaft system composed of couplings, a torquemeter, and motor dynamometers. The dynamic model of the gear system was constructed by including the mass, stiffness, and damping coefficients of all elements constituting the gear system, as shown in Fig. 4. At this stage, the commercial gear system analysis software Romax Nexus was used to configure the gear system model^16^. The dynamic behaviour of the system was confirmed by solving the equation of motion of the structural system presented in Eq. (1) based on the mass, stiffness, and damping of the developed model.

The gearbox components, such as gears, shafts, bearings, and housing, were reflected in the model based on the specifications of each element. The shaft was assumed to be a Timoshenko beam element to obtain shaft stiffness in the gear system model. Since the bearing stiffness exhibits nonlinear stiffness depending on the clearance, preload, and mounting method, it was linearized under the load conditions applied to the bearing and reflected in the system model^17^. The finite element (FE) tooth model was used for the meshing stiffness of the gear to consider the nonlinearity caused by system deflection and the elastic deformation of the gear teeth^4^.

The housing was modelled using a finite element approach as it has a complex three-dimensional shape. The information pertaining to the housing finite element model has been compiled in Table 3.

**Table 3:** Housing FEM model information.

Parameter	Housing LH	Housing RH	Rig
Element type	Tetra	Tetra	Tetra
Number of elements	166,621	157,199	174,214
Youngs modulus (GPa)	70.93	73.95	200
Density (kg/m^3^)	2660	2760	7850
Poisson’s ratio	0.3135	0.3107	0.3
Coefficient of expansive (μm/mC)	23.1	23.1	12.5

Mass and stiffness models were derived using the model reduction technique after connecting the hanging finite element model and bearing outer ring with the rigid body elements^18,19^. A finite-element model was developed to accurately implement the dynamic characteristics of the housing. This was enabled by performing an experimental modal analysis to compare and analyze the natural frequency and mode shape of the housing^15^. Table 4 shows the natural frequencies of the experimental mode analysis and simulation results. Using Eq. (6), an error was confirmed to occur within approximately 6% for the main mode of less than 3000 Hz. This confirms that the dynamic characteristics of the finite element model of the housing were similar to that of the test results.

**Table 4:** Comparison of natural frequencies between test and simulation.

Mode	1	2	3	4	5
Experiment (Hz)	1352.7	1720.5	2344.8	2539.0	2688.8
Simulation (Hz)	1311.0	1696.2	2211.4	2481.0	2580.4
Error (%)	− 3.1	− 1.4	− 5.7	− 2.3	− 4.0

6 $$Error=\frac{Simulation \;result-Experiment \;result}{Experiment \;result}\times 100$$

The system mass $\left[{{\varvec{M}}}_{S}\right]$, damping $\left[{{\varvec{C}}}_{S}\right]$, and stiffness $\left[{{\varvec{K}}}_{S}\right]$ matrices in the physical space can be obtained by combining the mass, stiffness, and damping of all elements used in the gear system. The dynamic behaviour of the system can be obtained by substituting these matrices into Eq. (3) and solving. However, the system stiffness and damping matrix have a non-diagonal component because the elements constituting a system are coupled to each other. Since the process of solving an equation including a matrix with an off-diagonal component requires a large amount of computation, each matrix must be decoupled and diagonalized so that it does not have an off-diagonal component. This separation and diagonalization is possible by transforming the system mass, damping, and stiffness matrices in the physical space into the modal space. The eigenvector matrix of the system is used as the transformation matrix. The amount of computation can be reduced by reducing the degrees of freedom by only including an appropriate number of modes^20^. The system damping was modelled using frequency-dependent proportional damping coefficients, as per prior research. It is known that damping is higher for low-frequency global modes and lower for high-frequency local modes^21^. Therefore, a damping value of 10% of critical damping was applied for the frequency range below 200 Hz, and Rayleigh damping was applied for the range above that frequency, with coefficients of $\alpha =600$ and $\beta =6\times {10}^{-6}$.

**Figure 5:**
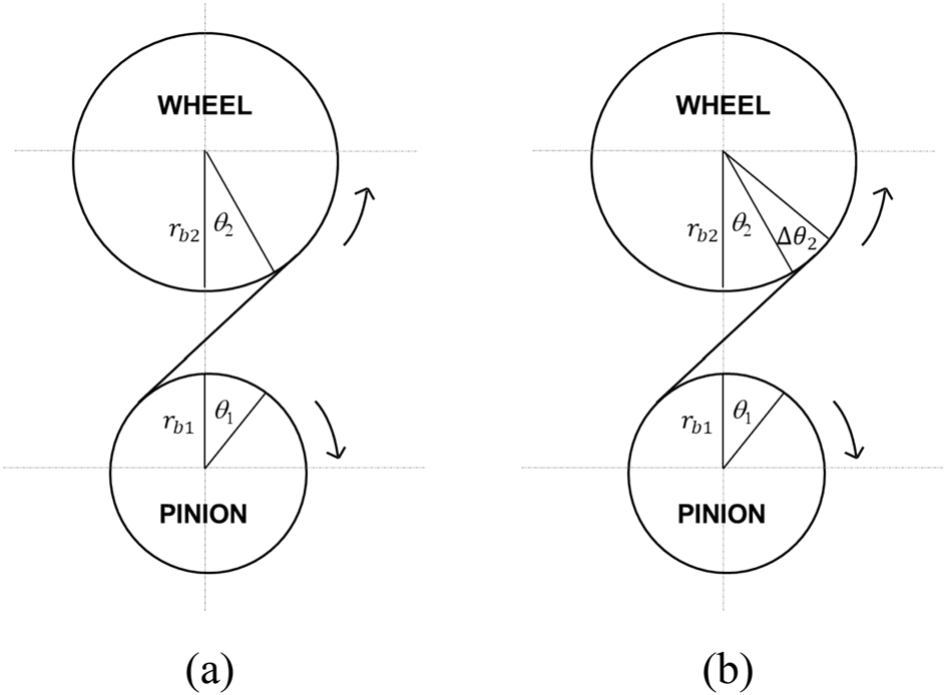
Gear motion diagram: (**a**) ideal gear pair and (**b**) actual gear pair.

In an ideal case, when the pinion rotates by ${\theta }_{1}$, the gear rotates by ${\theta }_{2}$; these two angles have the following relationship:

7 $${\theta }_{2}={\theta }_{1}\times \frac{{r}_{b1}}{{r}_{b2}},$$

where ${\theta }_{1}$ and ${\theta }_{2}$ are the rotation angles of the pinion and gear [mm], respectively, and ${r}_{b1}$ and ${r}_{b2}$ are the radii of the base circles of the pinion and gear [mm], respectively. However, as shown in Fig. 5, there is a difference between the actual and ideal gear rotation angle; this is caused by the variation in the gear mesh stiffness and gear misalignment. The actual gear rotation angle is expressed by

8 $${\theta }_{2}^{\mathrm{^{\prime}}}={\theta }_{2}+\Delta {\theta }_{2},$$

where ${\theta }_{2}^{\mathrm{^{\prime}}}$ is the actual gear rotation angle [deg.] and $\Delta {\theta }_{2}$ is the gear rotation angle deviation [deg.]. The transmission error of a gear is expressed as the difference in displacement between the rotation angle of the actual gear and that of the ideal gear on the line of action of the gear pair, as given by Eq. (9).

9 $$STE= {\theta }_{2}^{\mathrm{^{\prime}}}{r}_{b2}-{\theta }_{1}{r}_{b1}$$

**Figure 6:**
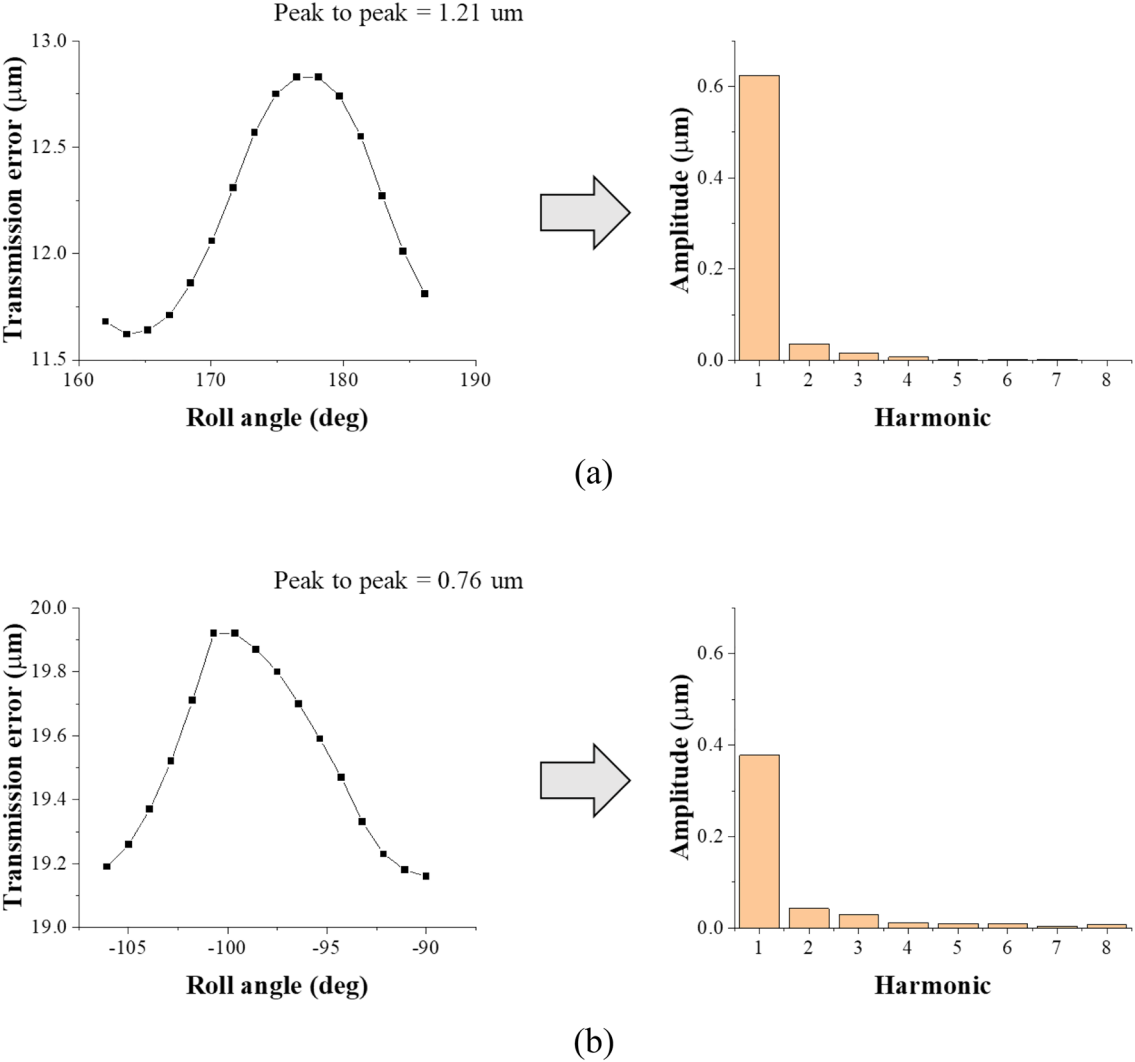
Transmission error of gear pair and its harmonics: (**a**) 1st stage and (**b**) 2nd stage.

Figure 6 shows the transmission error of the gear and the Fourier transform results obtained by the FE gear tooth model and the nonlinear contact model of the gear system model. The first harmonic was the most important excitation source, as the first harmonic component of the static transmission error of the first gear pair was the largest. Therefore, the first harmonic of the static transmission error occurring in the first gear pair was used as the excitation source for the gear system in this study.

The static transmission error of the gear, which is expressed as the displacement in the direction of the line of action, must be converted into an excitation force to excite the gear system. The static transmission error of the gear was converted into an excitation force by multiplying it by the average meshing stiffness of the gear pair, as shown in Eq. (10). The corresponding excitation force was applied to the pitch point of the gear in the direction of the line of action.

10 $${F}_{meshing}={k}_{average}\times {x}_{TE},$$

where ${F}_{meshing}$ is the gear mesh force [N], ${k}_{average}$ is the average gear mesh stiffness [N/mm], and ${x}_{TE}$ is the gear transmission error [mm].

### Vibro-acoustics model

**Figure 7:**
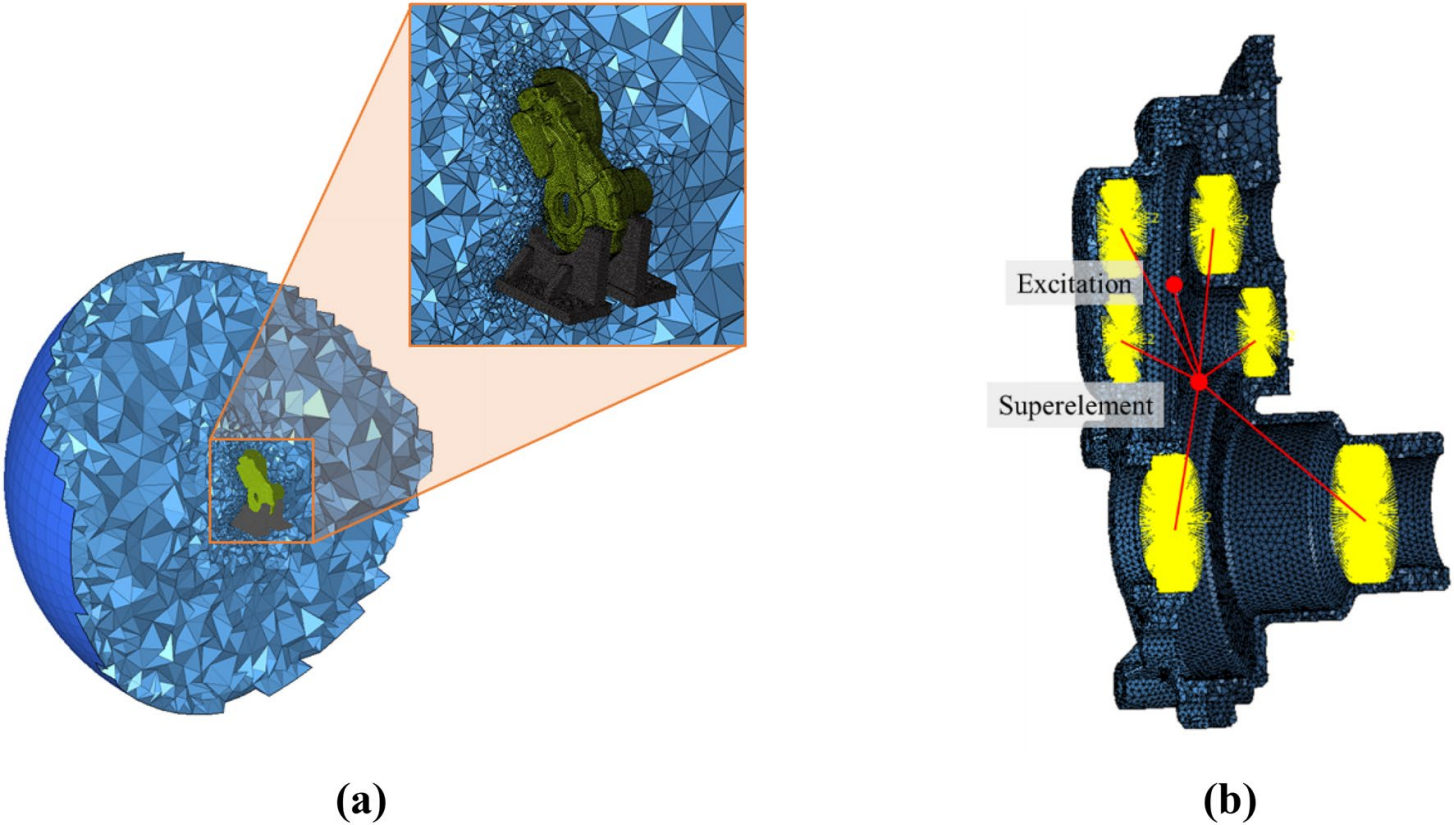
Finite element model used in the study: (**a**) acoustic cavity model and (**b**) finite element model with superelement.

The radiation noise of the gearbox is generated when the excitation force due to the transmission error of the gear is transmitted to the housing through the shaft and bearing, causing the housing to vibrate and interact with the surrounding fluid. The structural–acoustic coupled equation expressed in Eq. (5) must be solved using a numerical analysis method to realize this phenomenon analytically. A finite element sound field model was added to the previously developed gear system dynamic model, as shown in Fig. 7a, to obtain the sound pressure at the location of an installed microphone using structural–acoustic coupled numerical analysis. The modelling and numerical analysis were performed using the commercial finite element analysis software Altair Hypermesh and Optistruct^22^.

When performing acoustic analysis using a finite element model, the model must be configured to satisfy the Sommerfeld boundary condition. The Sommerfeld boundary condition is a condition in which sound waves propagate through the boundary of the sound field model without reflection and can be implemented using the PAABSF characteristic of NASTRAN^23^. Therefore, the Sommerfeld boundary condition was implemented in the analysis model by creating 2D elements surrounding the outer surface of the 3D sound-field element model, which reflects the air surrounding the housing. The PAABSF property was then assigned to these 2D elements.

A finite element model for housing optimization, as described earlier, is influenced by the internal transmission system. During structural–acoustic analysis, the transmission system is considered using either a system lumped parameter model or a finite element model. To mitigate the computational demands and time required for analysis, the entire transmission system was constructed as a system lumped parameter model within the vibration model. To incorporate the vibration model into the structural analysis software, NASTRAN's superelement was utilized.

A superelement was constructed by extracting mass and stiffness information, including gears, shafts, bearings, and couplings, but excluding housing, from a previously developed dynamic analysis model. Subsequently, the superelement was provided as input into the developed FE acoustic analysis model. In addition, as shown in Fig. 7b, the superelement was connected to the central node of the outer ring of the bearing, and the corresponding node was connected to the nodes located in the bearing attachment part of the housing finite-element model. Thus, all the loads and boundary conditions acting on the housing of the dynamic analysis model were considered.

Similar to the dynamic analysis, the gear meshing force calculated in Eq. (10) was the excitation source in the direction of the line of action at the pitch point of the first gear pair (excitation in Fig. 7b). The gear meshing force was transmitted to the housing through the superelement (shown in Fig. 7b), which excites the housing and causes it to vibrate. The vibration generated in the housing causes a fluctuation of the fluid elements at the interface between the housing and fluid elements, thereby generating sound pressure.

## Gearbox test and simulation results

**Figure 8:**
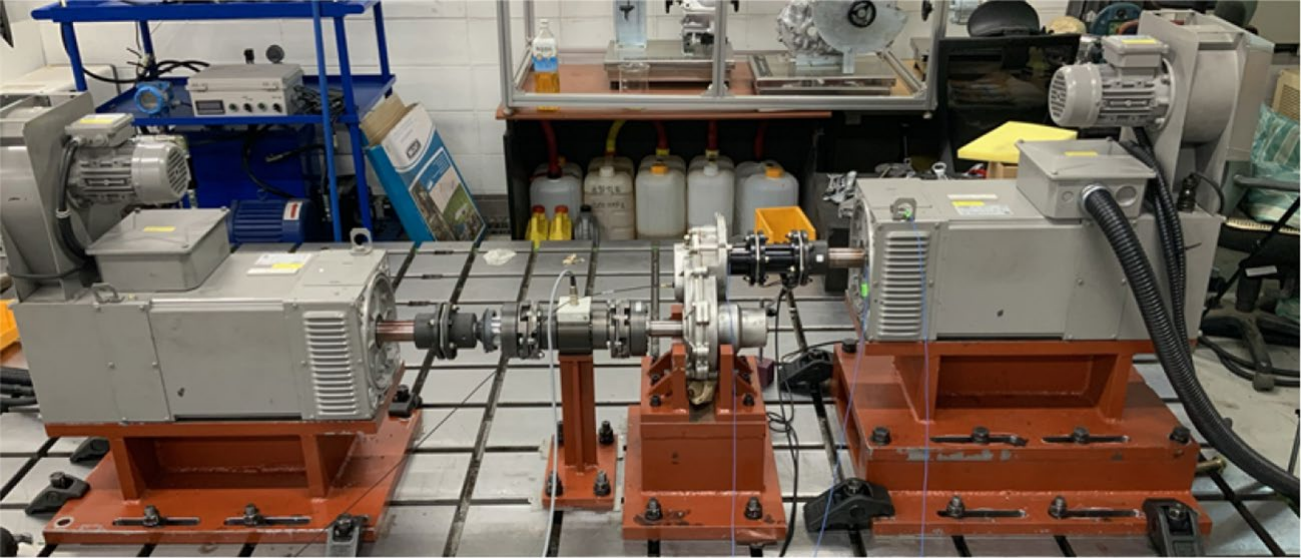
Photograph of the layout of the gearbox test.

The test equipment for measuring the vibration and noise of the gear system was constructed as shown in Fig. 2; the configured test equipment is shown in Fig. 8. The run-up test was performed at 0–3000 rpm, considering that the rate speed of the gearbox was 2650 rpm. The load of the output dynamometer was fixed at 22 Nm. The test conditions were set to satisfy 7 kW at a maximum speed of 3000 rpm during the increase test to evaluate the noise and vibration performance of the gearbox. The specifications of the sensors used in the test are summarized in Table 5.

**Table 5:** Specification of test equipment.

Equipment	Model	Specification
Torquemeter	HBM T22/1kNm	Nominal torque: 1 kNm Nominal speed: 20,000 rpm Output signal: $\pm$ 5 V and 10 $\pm$ 8 mA
Tachometer	PCB LT2	Speed range: 100,000 rpm
Microphone	PCB 378B02	Frequency range: 3.75–20,000 Hz Dynamic range: 137 dB re 20 μPa
Accelerometer	PCB 333B40	Sensitivity: 500 mV/g Frequency range: 0.5 to 3000 Hz

**Figure 9:**
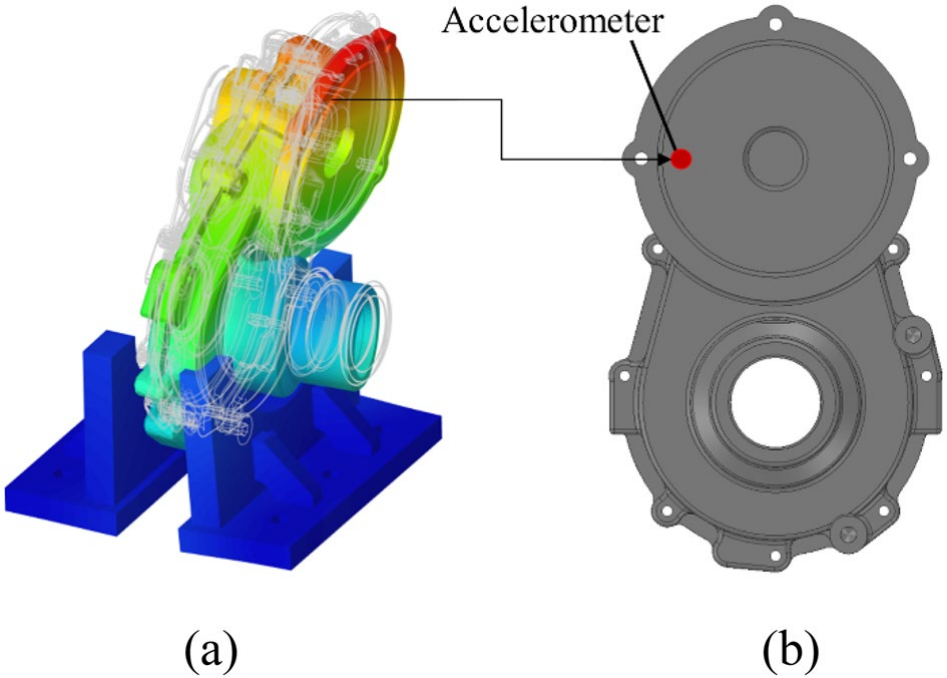
Modal analysis results: (**a**) housing mode shape and (**b**) accelerometer position.

An appropriate sensor location must be selected to obtain meaningful measurement results during the run-up test. The vibration response due to excitation does not appear clearly if the accelerometer is attached at a location where the behaviour of the housing is not well observed, making it difficult to verify the analysis result. Therefore, the accelerometer must be attached at a location where a large response is expected, such as a point where the relative displacement of the mode shape is large. In this study, a modal analysis was performed on the housing to select an appropriate location for the accelerometer. The mode shapes of the housing that appeared in the frequency domain of the run-up test were derived through modal analysis. The accelerometer location was selected based on these analysis results, which are shown in Fig. 9.

**Figure 10:**
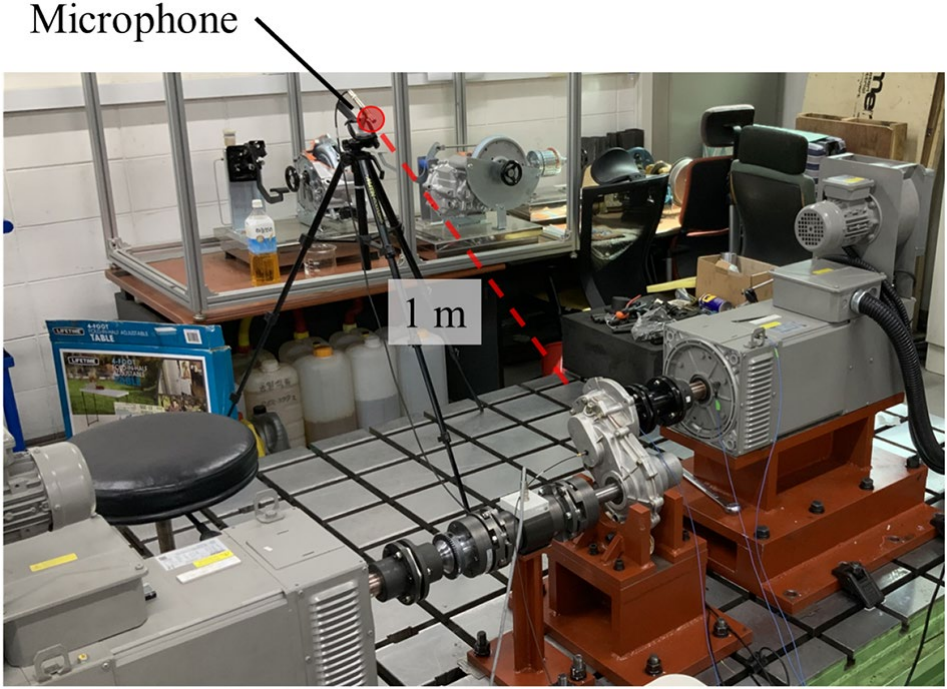
Microphone installation locations.

The microphone used to measure noise was installed 1 m from the test subject according to the ANSI/AGMA 6025 standard^24^. The detailed microphone installation location can be observed in Fig. 10. The microphone was installed in the direction where the gearbox installed in the vehicle predominantly radiates noise. The sound pressure level was calculated using Eq. (11) and an A-weighting filter was applied to the calculated result:

11 $$SPL=20\times {\text{log}}\left(\frac{P}{{P}_{ref}}\right),$$

where SPL is the sound pressure level [dB], $P$ is the measured sound pressure [Pa], and ${P}_{ref}$=20 μPa is the reference sound pressure [Pa].

Previously, the first harmonic of the first-stage gear pair was found to be the main excitation source in the gear system. Similarly, a run-up test confirmed that the first harmonic of the first-stage gear pair contributed the most to the vibration and noise^25^. In Tables 6 and 7, the ratios of vibration and noise magnitudes were presented. The sizes of different gear pair harmonics were expressed as ratios to the first harmonic of the first-stage gear pair at each speed and across the entire speed range. As evident from the tables, the first harmonic of the first-stage gear pair was predominant at the overall speed range, and it became more dominant as the speed increased. Gear noise exhibits low-frequency components at low speeds, and the magnitude of vibration and noise is more pronounced at higher speeds. Thus, this source was used as the target for validating and optimizing the simulation model.

**Table 6:** Vibration magnitude proportion of gear pairs.

Speed	1st-stage gear pair		2nd-stage gear pair	
	1st harmonic	2nd harmonic	1st harmonic	2nd harmonic
500 rpm	1.00	0.37	0.20	0.27
1000 rpm	1.00	2.83	0.40	3.00
1500 rpm	1.00	0.63	0.72	0.22
2000 rpm	1.00	0.22	0.58	0.63
2500 rpm	1.00	0.96	0.47	0.53
3000 rpm	1.00	0.93	0.09	0.25
Total speed range	1.00	0.98	0.51	0.57

**Table 7:** Sound pressure magnitude proportion of gear pairs.

Speed	1st-stage gear pair		2nd-stage gear pair	
	1st harmonic	2nd harmonic	1st harmonic	2nd harmonic
500 rpm	1.00	8.61	0.86	1.21
1000 rpm	1.00	4.34	0.11	1.88
1500 rpm	1.00	0.80	0.13	1.67
2000 rpm	1.00	0.45	0.14	0.33
2500 rpm	1.00	0.44	0.24	0.67
3000 rpm	1.00	0.66	0.71	0.25
Overall range	1.00	0.56	0.36	0.55

**Figure 11:**
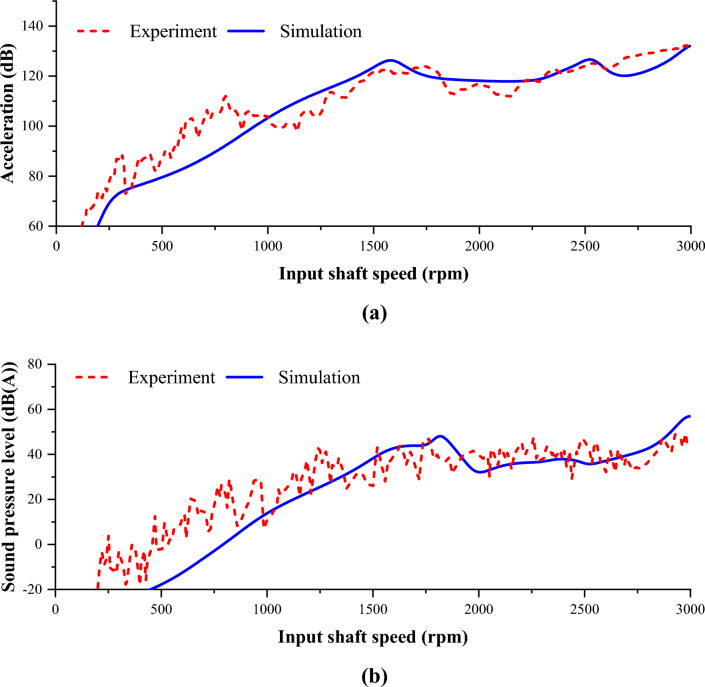
Results of experiment and simulation: (**a**) acceleration and (**b**) sound pressure level.

Figure 11 shows the validation results for the acceleration and sound pressure level. The validation was conducted through the first harmonic component of the first-stage gear pair, which is the target component of this study. Since the number of teeth of the first stage pinion is 14, the acceleration and sound pressure level expressed in Fig. 11 represent only the 14th order components of the overall data.

Figure 11a shows a comparison of the analysis and test results of the acceleration at the location of the accelerometer when the gear system model was excited through the first harmonic of the static transmission error of the first-stage gear pair. The analysis and test results generally showed similar tendencies. To evaluate them quantitatively, the root mean square of the vibration was calculated as follows.

12 $${a}_{rms}=\sqrt{\frac{1}{n}({a}_{1}^{2}+{a}_{2}^{2}+\dots +{a}_{n}^{2})}$$

Here, ${a}_{rms}$ is the root mean square of acceleration [m/s^2^] and ${a}_{n}$ is the acceleration at n Hz [m/s^2^]. According to the gear specification, the gear meshing frequency of the first harmonic for the first-stage gear pair at the maximum speed of 3000 rpm under the test condition was calculated to be 700 Hz. Thus, 700 was used as the value of n. Calculating the root mean square of the vibration level using Eq. (12) resulted in a test value of 114.70 dB and analysis value of 114.78 dB: an error of approximately 0.1 dB. Thus, the developed dynamic analysis model accurately reflects the actual vibration phenomenon.

Next, the sound pressure at the microphone position was calculated using the developed structural–acoustic coupled model. The structural–acoustic coupled system motion equation was numerically solved through structural analysis in the modal space, similar to the equation of motion of the structural system. Figure 11b shows a comparison of the SPL results from the analysis and test.

The root mean square of the test and analysis results calculated by Eq. (12) were 37.9 dB(A) and 37.7 dB(A), respectively: a difference of 0.2 dB(A). Consequently, the developed model was reliable for both vibration and noise.

## Optimization of gearbox housing shape

### Topology optimization

We used a topology optimization technique to optimize the shape of the housing. Topology optimization is a technique that determines the optimal material distribution within a design domain by optimizing the objective function according to a set of constrains^26,27^. We used the commercial software Altair Optistruct to implement topology optimization^22^.

An appropriate design domain must first be established to perform topology optimization. A simple initial structure must be created to establish the design domain when designing a new structure. However, a bulk shape should be added to the existing design shape when improving an existing design. Since numerical analysis is used during optimization, the design area must be discretized into finite elements. When a bulk shape is added to an existing design shape, the finite element model of the existing design shape and that of the newly added bulk shape must be connected through node sharing^15^.

The parameters for the design area should be set after designating the design area. The design parameter setting methods mainly used in topology optimization include homogenization^28^ and the solid isotropic material penalization (SIMP) method^29^. In this study, we used the SIMP method because it can express the design variables more simply than homogenization. In the SIMP method, the pseudomaterial density is specified as a design variable, and the material density varies continuously between 0 and 1:0 indicates a void state, and 1 indicates a completely solid state. The SIMP method applies a penalty function for the stiffness-density relationship, given by Eq. (13), to obtain a material density distribution between 0 and 1.

13 $$\widetilde{{\text{K}}}\left(\rho \right)={\rho }^{p}{\text{K}} 0\le {\rho }_{min}\le \rho \le 1,$$

where $\widetilde{\mathbf{K}}$ is the stiffness matrix of the penalized element, $\mathbf{K}$ is the stiffness matrix of the actual element, $\rho$ is the design variable density, ${\rho }_{min}$ is the minimum material density to prevent singularity, with ${\rho }_{min}$=0.001 used for this study^30^, and $p$ is the penalty coefficient, with $p$=2 used for this study^22^.

After setting the design area and variables, the objective function of optimization was numerically analyzed, and the optimization algorithms were implemented. Algorithms for topology optimization include sequential quadratic programming (SQP), the method of moving asymptotes (MMA), and dual optimizers based on separable convex approximations. The dual optimization method improved by Fleury and Braibant can obtain stable optimization results and is mainly used for structural optimization because of its low probability of convergence failure^31^. Therefore, an improved double-optimization method was used in this study.

### Optimal design for housing shape

**Figure 12:**
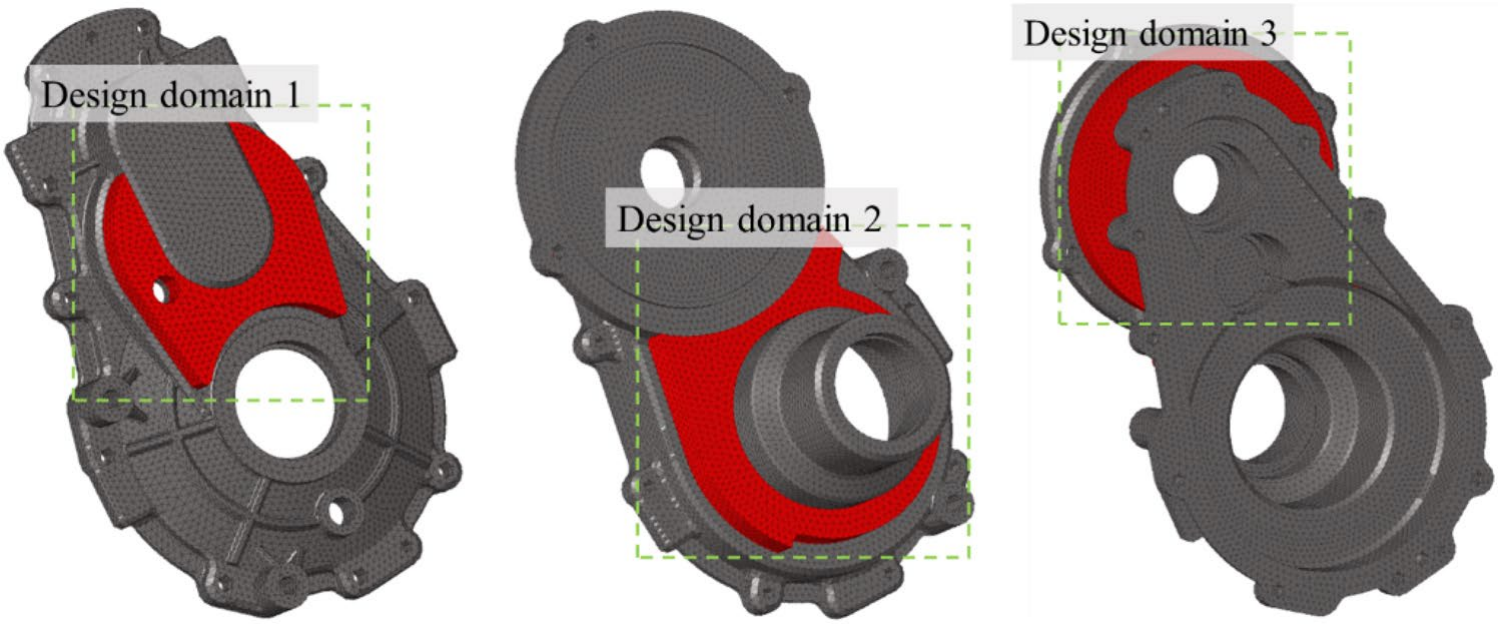
Three different design domains considered for topology optimization.

Three housing design areas were investigated, as shown in Fig. 12, considering the assembly of the agricultural UTV body, drive motor, and housing.

The optimization problem for the designated design area is formulated and expressed by Eq. (14):

14 $$\begin{aligned} {\text{minmax}}:\quad \quad & SPL = 20 \times {\text{log}}\left( \frac{p}{{{p_{ref}}}} \right), \\ {\text{subject to}}:\quad & V \leq {V_{max}}, \\ & {f_{min}}\left( { = 0Hz} \right) \leq freq \leq {f_{max}}\left( { = 700Hz} \right), \\ & \left[ {\begin{array}{*{20}{c}} {M_S}&0 \\ { - {S^T}}&{M_F} \end{array}} \right]\left\{ {\begin{array}{*{20}{c}} {\bar{w}} \\ {\bar{p}} \end{array}} \right\} + \left[ {\begin{array}{*{20}{c}} {C_S}&0 \\ 0&{C_F} \end{array}} \right]\left\{ {\begin{array}{*{20}{c}} {\dot w} \\ {\dot p} \end{array}} \right\} + \left[ {\begin{array}{*{20}{c}} {K_S}&{ - S} \\ 0&{K_F} \end{array}} \right]\left\{ {\begin{array}{*{20}{c}} w \\ p \end{array}} \right\} = \left\{ {\begin{array}{*{20}{c}} f \\ 0 \end{array}} \right\}, \\ \end{aligned}$$

where *V*: volume of design domain, mm3, *V*_max_: maximum volume of design domain, mm^3^.

$p$ represents the sound pressure analysis result at a single field point where the microphone is installed. Since the microphone was placed in the direction where the gearbox mounted on the vehicle primarily radiates nose, conducting topology optimization targeting this specific point holds significance.

The maximum allowable volume was set to 30% of that of the initial design area. Due to the gear transmission error occurring in the first-stage gear pair, the target frequency range for optimization was set from 0 to 700 Hz because the gear meshing frequency at 3000 rpm of the first harmonic is 700 Hz. The optimization problem was formulated using the minmax function to minimize the maximum value of the sound pressure at the microphone position that satisfies the structure-acoustic coupled equation of Eq. (5) in the set frequency range.

**Figure 13:**
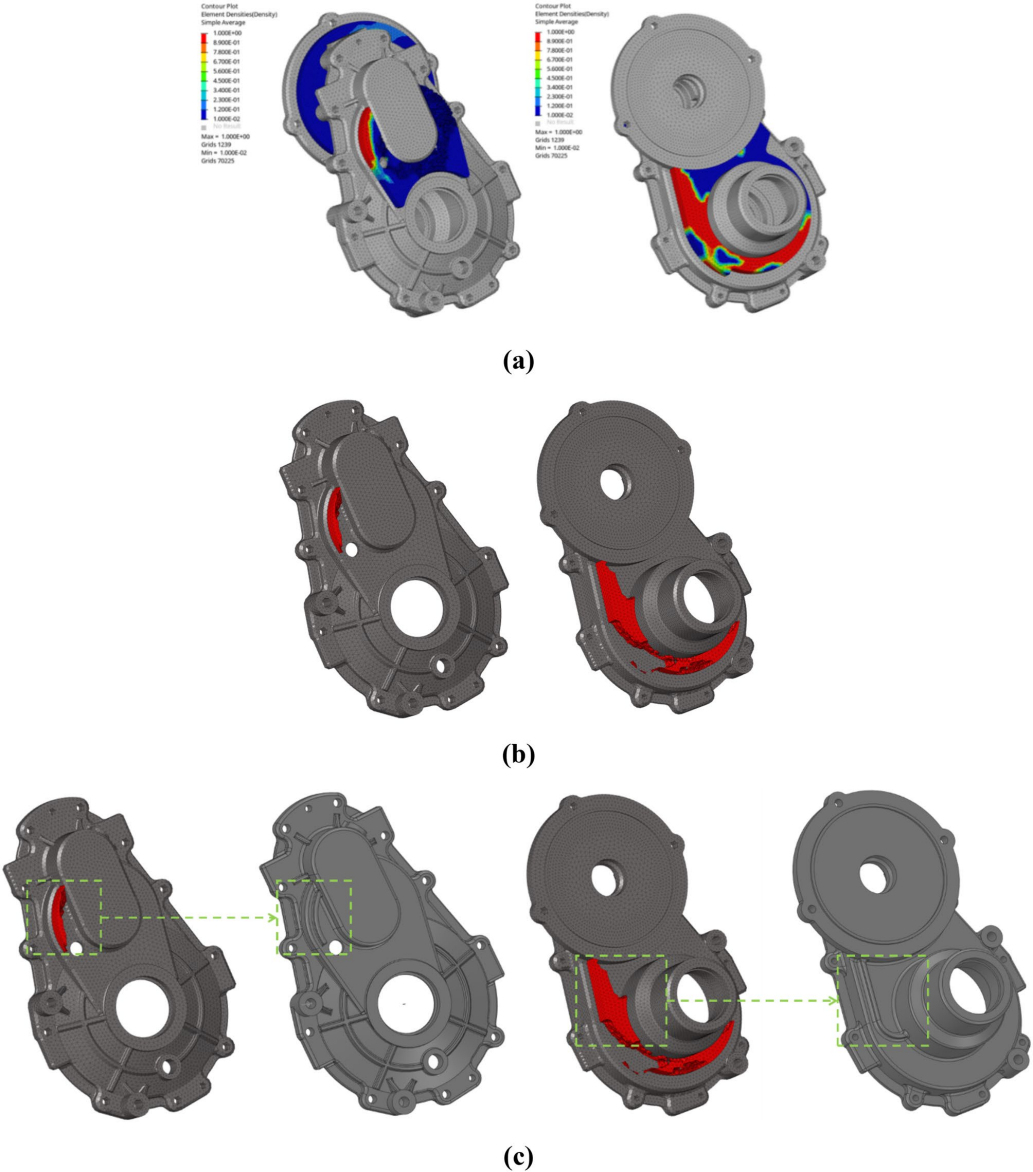
Optimization results: (**a**) Element density results of topology optimization, (**b**) Topology optimization design results, (**c**) Optimized housing shape.

The calculated element density distribution as a result of topology optimization is shown in Fig. 13a. The optimization results showed that Design domain 3 in Fig. 12 had no significant effect on reducing the sound pressure at the microphone position compared with Design domains 1 and 2.

In the optimization results, the user sets an appropriate threshold density to remove elements with a density lower than the threshold. Elements with a density greater than or equal to the threshold density can be made into completely filled elements. The material distribution of the final design area is derived through this process, and is shown in Fig. 13b.

Since the additional rib design area for the housing reinforcement design was identified through the topology optimization result, a rib shape design was implemented in the relevant area. The ribs were designed to consider the manufacturability and assembly of the housing, and the final optimized housing shape is shown in Fig. 13c. The weights of the housing before and after optimization were 4.836 kg and 4.847 kg, respectively, which is a difference of less than 0.2%.

**Figure 14:**
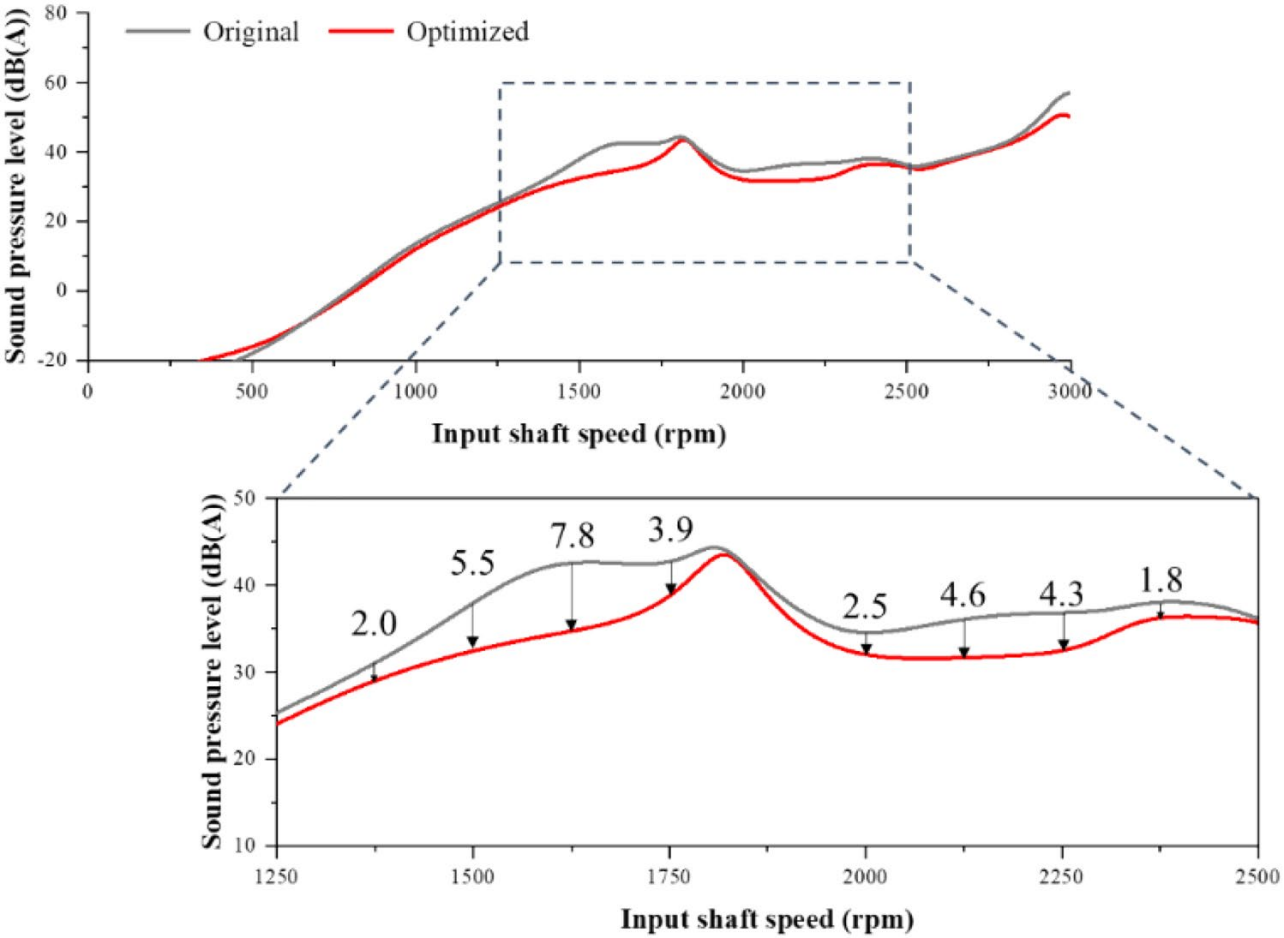
Comparison of SPL results before and after optimization.

After replacing the housing used in the existing structural–acoustic coupled model with an optimized housing, sound pressure analysis was performed again. Figure 14 shows the results of the sound pressure analysis at the microphone position from the excitation of the first harmonic of the first-stage gear pair before and after the optimal housing design. The optimal housing design reduced radiated noise in all areas, except for the low-speed area. The effect of reducing the radiated noise was judged to be effective as the radiated noise in the low-speed section was less than 0 dB and was not in the audible sound pressure range.

As a result of the optimization, the noise level was reduced by approximately 3.46 dB(A) in the main operating area at 1250–2500 rpm of the agricultural UTV and by approximately 2.43(A) in the entire area based on the root mean square calculated by Eq. (12). This is approximately a 9.2% and 6.8% reduction, respectively, compared to the existing radiated noise in each area; hence, the noise reduction effect is excellent.

## Conclusion

This study used analysis, testing, and optimization to reduce the radiated noise of a gearbox by modifying its housing shape. The process and results of each step are summarized as follows.

1. A dynamic analysis model of a gear system and a radiation noise prediction model were developed to optimize the shape of the gearbox housing. The dynamic analysis model was constructed to include the physical properties of all the elements used for the model validation test. Structures such as gearbox housings radiate structural noise through the excitation of the surrounding fluid due to vibration. Thus, a structural–acoustic coupled analysis that predicts structural-acoustic behaviour is required to accurately analyze this phenomenon. Therefore, a finite element model of the sound field around the housing was developed and used for noise analysis to perform structural–acoustic coupled analysis.
2. The test equipment was configured to have the same structure as that used in the simulation model. The gearbox run-up test and analysis results of vibration and noise generated in the gearbox housing showed errors within 0.1 dB and 0.2 dB(A), respectively. Thus, the model was reliable.
3. The housing was optimized using topology optimization using the verified analysis model. The objective function was set to minimize the sound pressure to reliably reduce the radiation noise. The topology optimization determined a housing topology that reduced the radiation noise. A rib was designed and placed in the housing based on this optimization result. Structural-acoustic coupled analysis was performed by attaching the optimally designed housing to the previously developed analysis model. The radiated noise was reduced by approximately 2.43 dB(A) for the entire operating speed range.

## Data Availability

The data that support the findings of this study are available from the corresponding author upon reasonable request.
